# The non-linear association of physical fatigue with depression and anxiety among mental health professionals who recovered from COVID-19 infection: a national survey in China

**DOI:** 10.3389/fpsyt.2025.1610910

**Published:** 2025-08-22

**Authors:** Yu-Cheng Wang, Yi-Ran Huang, He-Li Sun, Yuan Feng, Pan Chen, Zhaohui Su, Teris Cheung, Gabor S. Ungvari, Lloyd Balbuena, Feng-Rong An, Yu-Tao Xiang, Gang Wang

**Affiliations:** ^1^ Unit of Psychiatry, Department of Public Health and Medicinal Administration, and Institute of Translational Medicine, Faculty of Health Sciences, University of Macau, Macau, Macao SAR, China; ^2^ Centre for Cognitive and Brain Sciences, University of Macau, Macau, Macao SAR, China; ^3^ Beijing Key Laboratory of Mental Disorders, National Clinical Research Center for Mental Disorders & National Center for Mental Disorders, Beijing Anding Hospital, Capital Medical University, Beijing, China; ^4^ School of Public Health, Southeast University, Nanjing, China; ^5^ School of Nursing, Hong Kong Polytechnic University, Hong Kong, Hong Kong SAR, China; ^6^ Section of Psychiatry, University of Notre Dame Australia, Fremantle, WA, Australia; ^7^ Division of Psychiatry, School of Medicine, University of Western Australia, Perth, WA, Australia; ^8^ Department of Psychiatry, University of Saskatchewan, Saskatoon, SK, Canada

**Keywords:** COVID-19, physical fatigue, depression, anxiety, mental health professionals, nonlinear association

## Abstract

**Background:**

The COVID-19 pandemic took a toll on everyone’s health and mental health professionals were no exception. This study examined the trajectory of the relationship between levels of physical fatigue and each of depression and anxiety in mental health professionals (MHPs) recovering from COVID-19.

**Methods:**

A national survey of 9,858 MHPs who had recovered from COVID-19 was conducted between January and February 2023. The nine-item Patient Health Questionnaire (PHQ-9), the 7-item Generalized Anxiety Disorder (GAD-7) scale, and a numerical rating scale were used to measure depression, anxiety and physical fatigue, respectively. Logistic regression with restricted cubic spline (RCS) models were created to examine the association of physical fatigue with depression and anxiety.

**Results:**

The prevalence of depression and anxiety in MHPs who recovered from COVID-19 infection were 47.0% (95%CI: 46.0-48.0%) and 28.9% (95%CI: 28.0-29.8%) respectively. The prevalence of moderate to severe physical fatigue was 44.2% (95%CI: 43.2-45.2%). The RCS models revealed a significant nonlinear relationship between physical fatigue and both depression and anxiety, with an inflection point at a fatigue score of 4. Above this threshold, the risk of both conditions increased significantly. Participants with poor perceived health and lower socioeconomic status had a significantly greater increase in depression and anxiety when fatigue levels were higher.

**Conclusions:**

Moderate to severe physical fatigue was associated with depression and anxiety in MHPs recovering from COVID-19. Interventions aimed at alleviating fatigue may play a critical role in improving mental health outcomes in this vulnerable population.

## 1 Introduction

The Coronavirus Disease 2019 (COVID-19) pandemic had an unprecedented and widespread impact on healthcare systems globally, imposing physical and psychological burdens for healthcare workers. Mental health professionals (MHPs) were at the forefront of the response to the mental health problems brought about by COVID-19. As a result, MHPs were faced with stressors such as extended work hours, increased patient numbers, and concerns about infection (1). Apart from treating patients under uncontrollable circumstances, many MHPs themselves were infected with COVID-19 or lived with the fear of being infected (2).

The distress experienced by MHPs infected with COVID-19 increased their risk of developing anxiety and depression (3–5). A recent umbrella review estimated that 24.83% (95% CI:21.41-28.25) of MHPs suffered from depression and 24.94% (95% CI:21.83-28.05) from anxiety (6). Therefore, it is crucial to examine the mental health status of MHPs, including those recovering from COVID-19 in the post-pandemic period.

COVID-19 sequelae frequently present with pronounced physical manifestations, of which physical fatigue is one of the most common and may have distinct pathophysiological mechanisms and psychological impact (7, 8). Physical fatigue refers to a subjective feeling of tiredness, lack of energy, and physical exhaustion that interferes with daily activities (9, 10). Physical fatigue can be classified as mild (i.e., minimal impact on daily activities), moderate (i.e., significantly limiting daily activities) or severe (i.e., preventing most activities) based on its intensity and impact.

Post-infection fatigue may persist for several months, severely affecting the individual’s mental health (7). This relationship can be explained by various theories. For instance, the diathesis-stress model suggests that biological vulnerabilities could interact with environmental stressors to precipitate psychological symptoms when a threshold is exceeded (11). Physical fatigue in this case acts as both a chronic stressor and biological vulnerability, which overwhelms adaptive capacity and contributes to depression and anxiety when a threshold is reached. Furthermore, post-COVID physical fatigue could affect mental health through physiological pathways associated with cognitive impairment such as autonomic nervous system dysfunction (12). From a clinical perspective, multiple studies have established a clear association between fatigue and mental health outcomes, particularly depression and anxiety (13, 14). Fatigue was a central symptom of depression during the pandemic (15). A meta-analysis of 13 cross-sectional studies covering 33,062 healthcare professionals (1) revealed that many healthcare professionals had increased rates of anxiety and depression, both of which were strongly associated with higher fatigue levels and decreased physical and mental well-being. Fatigue has both direct and indirect effects on mental health. It can impair daily functioning, reduce productivity, and limit social engagement, all of which are known risk factors for depression (16–18). Additionally, fatigue often exacerbates symptoms of anxiety through its association with disrupted sleep patterns, cognitive impairments, and heightened physiological arousal (19, 20). These effects create a cycle that worsens psychological distress.

While the relationship between physical fatigue and mental health outcomes is well documented, it may not follow a simple linear pattern. As fatigue increases, the likelihood of suffering from depression and anxiety may increase disproportionately in excess of a certain threshold (21, 22). To date, no studies about potential non-linear associations of physical fatigue with depression or anxiety among MHPs have been published. Restricted cubic spline (RCS) models are a widely used method for examining if a non-linear relationship holds between variables (22).

To fill this gap, the present work examined the prevalence and trajectory of depression and anxiety with fatigue in MHPs recovering from COVID-19. Based on theoretical frameworks (11) and existing literature (13, 14), this study hypothesized that there would be a non-linear relationship between physical fatigue and depression and anxiety; MHPs who reported having fatigue beyond a certain threshold would be at substantially higher odds of anxiety and depression.

## 2 Methods

### 2.1 Study design and participants

The data came from a national cross-sectional survey that was carried out with the help of the panel members of the Chinese Society of Psychiatry and the Psychiatry Branch, Chinese Nursing Association immediately following the cessation of China’s Dynamic Zero-COVID Policy (January to February 2023). Given the risk of COVID-19 transmission, this study adopted an online snowball sampling method to ensure the safety of participants, as previous studies have done (23, 24). The survey was administered via the WeChat-based Questionnaire Star platform, a widely used and secure online survey program. A Quick Response (QR) code linked to the study invitation and assessment instruments was distributed to all public psychiatric hospitals nationwide, allowing for convenient and anonymous participation. This approach was chosen to maximize response rates while minimizing participant burden and potential biases associated with face-to-face data collection. To be eligible, prospective participants had to be (1)18 years of age or older; (2) employed as mental health professionals (e.g., doctors, nurses and nursing assistants) in psychiatric hospitals or psychiatric departments of general hospitals in China during the survey period; (3) have recovered from COVID-19 infection; and (4) able to understand Chinese and provide written informed consent. The study protocol was approved by the Institutional Review Board (IRB) of Beijing Anding Hospital, China (2020 – Keyan (No. 10)) based on the local ethical regulations and the Declaration of Helsinki.

### 2.2 Measures

The socio-demographic and clinical characteristics of participants were collected, including age, gender, marital status, perceived health and economic status, as well as self-reported smoking and drinking behavior.

Depression level was assessed using the validated Chinese version of the Patient Health Questionnaire (PHQ-9), which contains nine items and has good validity and reliability among health workers (25, 26). Each item was scored on a 4-point Likert scale ranging from 0 (“not at all”) to 3 (“nearly every day”). Total scores range from 0 to 27, with a higher score indicating more severe symptoms. A cut-off score of 5 was also used to classify people as having depressive symptoms, as recommended by a previous study (27).

Anxiety level was assessed using the validated Chinese version of the Generalized Anxiety Disorder (GAD-7) scale with seven items (28, 29). Participants were asked to report the frequency of anxiety-related symptoms. Each item was rated on a 4-point Likert scale ranging from 0 (“not at all”) to 3 (“nearly every day”), with total scores ranging from 0 to 21. A cut-off score of 5 was applied to define having anxiety symptoms (27).

Physical fatigue was assessed using a numerical rating scale (NRS), a widely validated and standardized tool for measuring physical fatigue (30, 31). Participants were asked the following question: “What is the number that best matches your current level of physical fatigue?” Responses were recorded on an NRS scale ranging from 0 to 10, where 0 indicated “no fatigue” and 10 represented “extreme fatigue” (30). For the purpose of logistic regression analysis (see below), physical fatigue was categorized into two groups: mild fatigue or below (NRS score 0–5) and moderate to severe fatigue (NRS score 6–10) (32).

### 2.3 Statistical analysis

All analyses were performed using R software version 4.3.2 (33). The RCS models were implemented using the rms package (Version 6.8.2) (34). The normality of distribution for continuous variables was tested using the Kolmogorov-Smirnov test. Group differences in socio-demographic and clinical characteristics between participants with depression or anxiety and those without were examined using independent t-tests, Mann-Whitney U test, and chi-square tests, as appropriate.

Binary logistic regression analysis with the ‘enter’ method was conducted to investigate the associations between physical fatigue and depression or anxiety, adjusting for confounders identified in the univariate analyses (p < 0.05). These confounders were age, gender, marital status, perceived economic status, and COVID-19 infection history. Physical fatigue level (categorized as no and mild, moderate and severe) was the independent variable and either depression or anxiety was the dependent variable. Multicollinearity was examined with variance-inflation factors (VIF). A p-value of <0.05 was considered statistically significant across all analyses (two-tailed).

To test our hypothesis of nonlinearity, restricted cubic spline (RCS) models (22) were employed, with having depression and anxiety (treated as dichotomous variables) as separate dependent variables and fatigue (treated as a continuous variable) as the independent variable. RCS models allow for a flexible assessment of nonlinear relationships by fitting smooth curves to the data, while maintaining linearity beyond the boundary knots (35). First, nonlinearity was assessed through a formal nonlinearity test. Following confirmation of nonlinearity, the number of knots was determined based on model fit criteria, specifically the Akaike Information Criterion (AIC) and Bayesian Information Criterion (BIC). A model with 3 interior knots was selected as it provided the optimal balance between goodness of fit and model complexity. Knots serve as pivot points where the curve can change direction to better fit the data, and the points where the slope changes significantly are referred to as inflection points. This approach offers a detailed and informative depiction of the relationship between fatigue and mental health outcomes (21).

Subgroup analyses were conducted to assess whether gender, marital status, perceived economic status, perceived health status, smoking, drinking, and COVID-19 quarantine experience modified the relation of fatigue and depression or anxiety.

## 3 Results

### 3.1 Participant characteristics

A total of 11,218 mental health professionals were invited to participate in this study, of whom 9,858 met the eligibility criteria and were included for analyses. Among them, 4,640 (47.0%, 95% CI: 46.0-48.0%) reported depression (PHQ-9 total score ≥ 5), 2,857 (28.9%, 95% CI: 28.0-29.8%) reported anxiety (GAD-7 total score ≥ 5), and 4,364 (44.2%, 95%CI: 43.2-45.2%) reported moderate to severe physical fatigue (NRS score ≥ 6). The demographic and clinical characteristics of the study sample and the results of univariate analyses are presented in **Table 1**.

**Table 1:** Demographic and clinical characteristics of mental health professional.

Variables	Total	Depressive symptoms (PHQ-9>=5)		Univariate analysis		Anxiety symptoms (GAD-7>=5)		Univariate analysis	
		Yes (N=4,640)	No (N=5,218)			Yes (N=2,857)	No (N=7,001)		
	n (%)	n (%)	n (%)	*χ* ^2^	p	n (%)	n (%)	*χ* ^2^	p
Male	1,750 (17.7)	855 (18.4)	895 (17.1)	2.7	0.09	553 (19.3)	1,197 (17.1)	7.0	**0.008**
Married	7,209 (73.1)	3,418 (73.6)	3791 (72.6)	1.2	0.25	2,109 (73.8)	5,100 (72.8)	0.9	0.32
Perceived economic status									
Poor	1,067 (10.8)	729 (15.7)	338 (6.6)	291.6*	**<0.001**	513 (17.9)	554 (7.9)	246.1*	**<0.001**
Normal	8,188 (83.0)	3,745 (80.7)	4443 (85.1)			2,246 (78.6)	5,942 (84.8)		
Good	603 (6.1)	166 (3.5)	437 (8.3)			98 (3.4)	505 (7.2)		
Perceived health status									
Poor	653 (6.6)	579 (12.4)	74 (1.4)	1208.6*	**<0.001**	438 (15.3)	215 (3.0)	882.5*	**<0.001**
Normal	7,043 (71.4)	3,653 (78.7)	3390 (64.9)			2,215 (77.5)	4,828 (68.9)		
Good	2,162 (21.9)	408 (8.7)	1754(33.6)			204 (7.1)	1,958 (27.9)		
Being quarantined during the COVID-19 pandemic	5,455 (55.3)	2,769 (59.6)	2686 (51.4)	66.8	**<0.001**	1,705 (59.6)	3,750 (53.5)	30.6	**<0.001**
Smoking	947 (9.6)	525 (11.3)	422 (8.0)	29.4	**<0.001**	352 (12.3)	595 (8.4)	34.1	**<0.001**
Drinking	1,857 (18.8)	1,060 (22.8)	797 (15.2)	92.0	**<0.001**	691 (24.1)	1,166 (16.6)	75.2	**<0.001**
Physical Fatigue level									
No and mild fatigue	5,494 (55.7)	1,489 (32.0)	4005 (76.7)	1985.7	**<0.001**	685 (23.9)	4,809 (68.6)	1644.2	**<0.001**
Moderate and severe fatigue	4,364 (44.2)	3,151 (67.9)	1213 (23.3)			2172 (76.0)	2,192 (31.3)		
	Mean (SD)	Mean (SD)	Mean (SD)	Z	p	Mean (SD)	Mean (SD)	Z	p
Age	34.8 (8.3)	35.1 (8.0)	34.5 (8.5)	-4.9	**<0.001**	35.0 (7.9)	34.7 (8.4)	-3.0	**0.003**
Work years	12.6 (9.0)	13.0 (8.7)	12.3 (9.3)	-5.8	**<0.001**	12.9 (8.7)	12.5 (9.2)	-3.7	**<0.001**
QOL	6.1 (1.5)	5.2 (1.2)	6.8 (1.4)	51.4	**<0.001**	5.0 (1.2)	6.5 (1.4)	44.2	**<0.001**
Physical Fatigue	3.9 (2.4)	5.3 (2.1)	2.7 (2.0)	-51.9	**<0.001**	5.7 (2.1)	3.2 (2.1)	-46.6	**<0.001**

Bolded values: <0.05; PHQ-9, Patient Health Questionnaire-9 items; GAD-7, General Anxiety Disorder-7 items; QOL, Quality of life; SD, standard deviation; *: degree of freedom=2, others=1.

### 3.2 Multivariate logistic regression analyses

**Figure 1:**
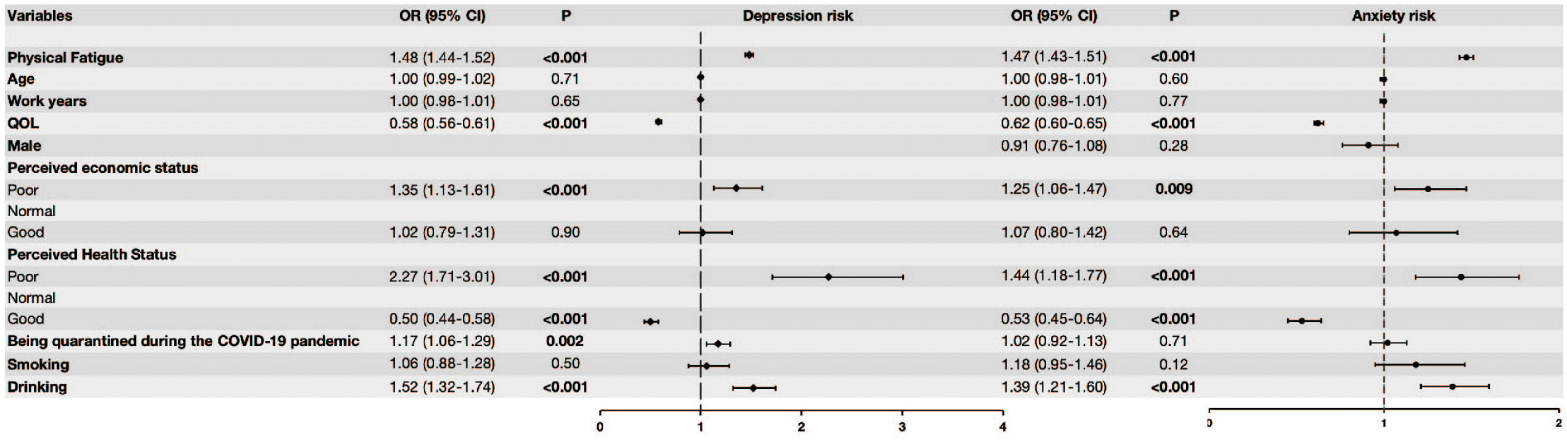
Logistic regression models of physical fatigue and depression, and anxiety. Model 1: Logistic regression models of physical fatigue and depression after adjusting for age, work years, quality of life, perceived economic status, perceived health status, being quarantined, smoking and drinking. Model 2: Logistic regression models of physical fatigue and anxiety after adjusting for age, work years, quality of life, perceived economic status, perceived health status, gender, being quarantined, smoking and drinking.

The multivariate logistic regression model showed that physical fatigue was a risk factor for both the presence of depression and anxiety (**Table 2**, **Figure 1**). After adjusting for potential confounders (e.g., age, work years, quality of life, perceived economic status, perceived health status, gender, being quarantined, smoking and drinking) higher levels of physical fatigue were associated with significantly increased risk of depression (OR = 1.48, 95% CI: 1.44–1.52, p < 0.001) and anxiety (OR = 1.47, 95% CI: 1.43–1.51, p < 0.001). The results showed that there was no multicollinearity. The VIFs were in the range: 1.01-7.43 for depression, and 1,01-7.47 for anxiety (**Supplementary Table S1**). Additionally, age, work years, gender and smoking status were not significantly associated with either depression or anxiety in the fully adjusted models.

**Table 2:** Logistic regression models of physical fatigue and depression, and anxiety.

Variables	Depressive symptoms (a)			Anxiety symptoms (b)		
	p	OR	95% CI	p	OR	95% CI
Physical Fatigue	**<0.001**	1.48	1.44-1.52	**<0.001**	1.47	1.43-1.51
Age	0.71	1.00	0.99-1.02	0.60	1.00	0.98-1.01
Work years	0.65	1.00	0.98-1.01	0.77	1.00	0.98-1.01
QOL	**<0.001**	0.58	0.56-0.61	**<0.001**	0.62	0.60-0.65
Male	–	–	–	0.28	0.91	0.76-1.08
Perceived economic status						
Poor	**<0.001**	1.35	1.13-1.61	**0.009**	1.25	1.06-1.47
Normal	–	–	–	–	–	–
Good	0.90	1.02	0.79-1.31	0.64	1.07	0.80-1.42
Perceived health status						
Poor	**<0.001**	2.27	1.71-3.01	**<0.001**	1.44	1.18-1.77
Normal	–	–	–	–	–	–
Good	**<0.001**	0.50	0.44-0.58	**<0.001**	0.53	0.45-0.64
Being quarantined during the COVID-19 pandemic	**0.002**	1.17	1.06-1.29	0.71	1.02	0.92-1.13
Smoking	0.50	1.06	0.88-1.28	0.12	1.18	0.95-1.46
Drinking	**<0.001**	1.52	1.32-1.74	**<0.001**	1.39	1.21-1.60

QOL, Quality of life.

(a): adjusted for age, work years, quality of life, perceived economic status, perceived health status, being quarantined, smoking, drinking.

(b): adjusted for age, work years, quality of life, perceived economic status, perceived health status, gender, being quarantined, smoking, drinking. Bold values indicate statistically significant results (p < 0.05).

### 3.3 Nonlinear associations between physical fatigue and depression and anxiety

**Figure 2:**
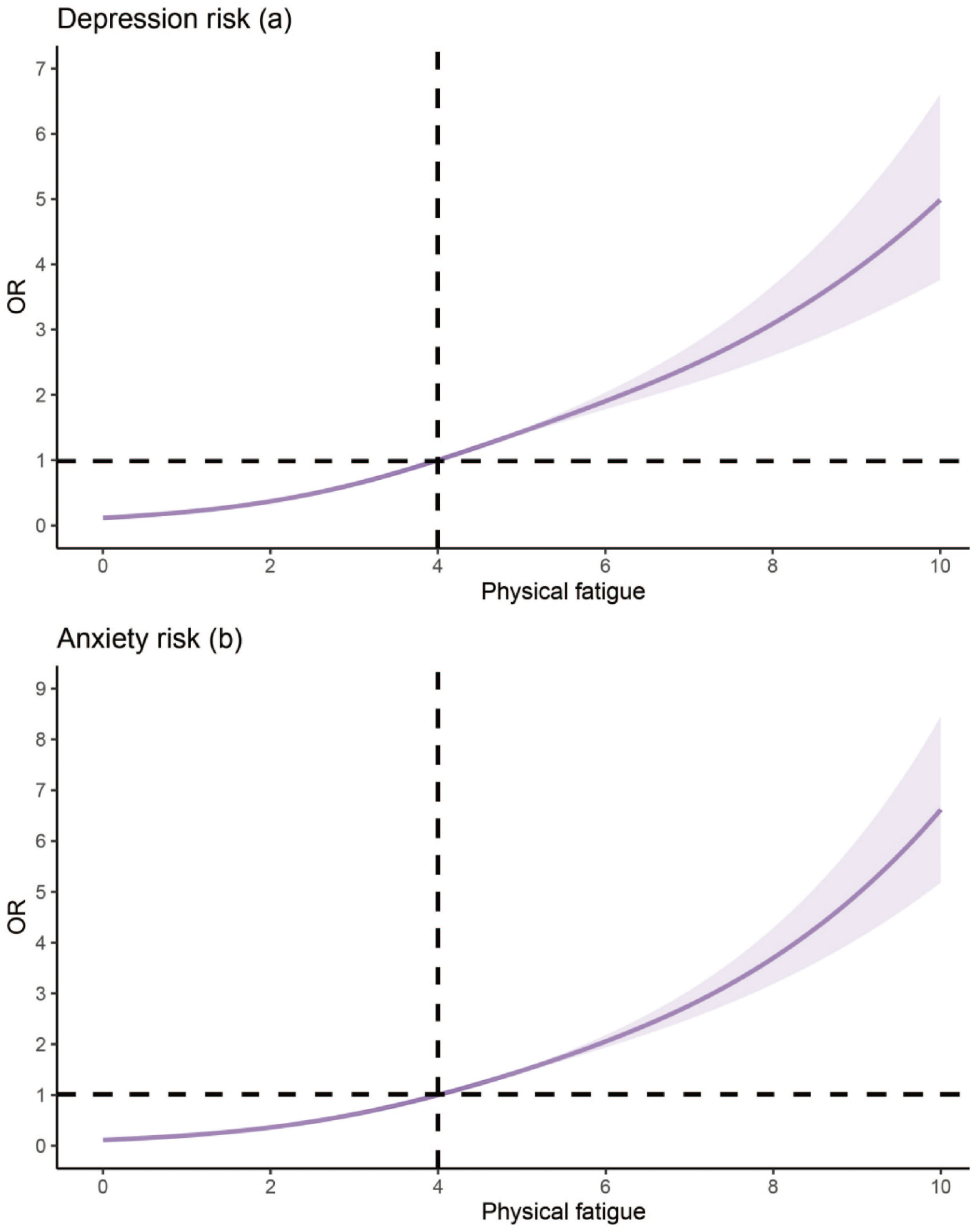
Nonlinear association between depression **(a)**, anxiety **(b)** and physical fatigue. Vertical line indicates inflection point.

The RCS models revealed a significant nonlinear relationship for physical fatigue and presence of depression and anxiety, with an inflection point occurring at a fatigue score of 4 in both models. For depression, a significant nonlinear relationship was observed (p for nonlinearity < 0.001), and the model fitted with 3 knots provided the best fit (AIC = 9413.169, BIC = 9506.717). As shown in **Figure 2a**, the risk of depression remained relatively flat at lower fatigue levels (0–3), followed by a distinct inflection point at a fatigue score of 4, which indicated that participants above this threshold have an accelerated risk of depression.

Similarly, the RCS model for anxiety demonstrated a significant nonlinear association (p for nonlinearity < 0.001), with the model fitted with 3 knots yielding the best fit (AIC = 8662.678, BIC = 8763.423). There was a significant increase in the risk of anxiety occurring at fatigue levels above the infection point of 4 (p < 0.001, **Figure 2b**). The increase in anxiety risk became particularly pronounced at the threshold of 4 and above, emphasizing the compounding mental health burden of moderate to severe fatigue in this population.

**Figure 3:**
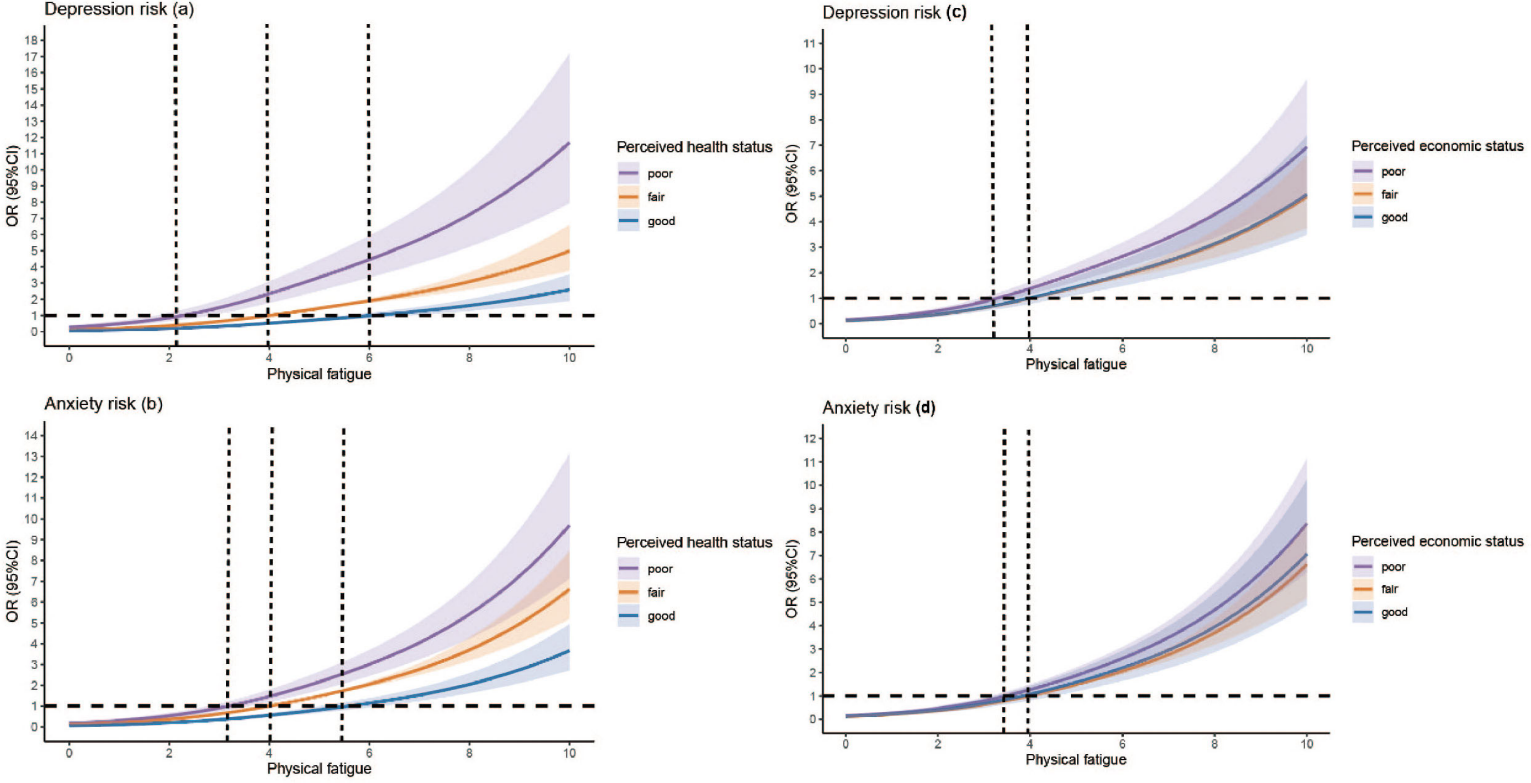
Nonlinear association between depression **(a, c)**, anxiety **(b, d)** and physical fatigue, stratified by perceived health status and perceived economic status. Vertical lines indicate inflection points.

Subgroup analyses revealed significant variations in the relationship between physical fatigue and depression/anxiety by health and economic status. Participants with poor perceived health experienced a substantially stronger association between fatigue and depression compared to those with good health. In contrast, participants with good health maintained a relatively lower risk of depression, even at higher fatigue levels. The inflection points for fatigue were at 2, 4, and 6 for the poor health status group, normal health status group, and good health status group, respectively (**Figure 3**).

Similarly, the relationship between fatigue and anxiety was significantly more pronounced among participants with poor perceived economic status. Those in the poor economic status group exhibited a steeper increase in anxiety risk compared to participants with normal or good economic status (**Figure 3**). This emphasizes the amplifying effect of economic status on the mental health impact of fatigue. The results on the subgroup analyses are presented in **Supplementary Figures S1-S6**.

## 4 Discussion

This was the first study to examine the nonlinear relationship between physical fatigue and depression and anxiety for MHPs recovering from COVID-19 infection. The main finding is that physical fatigue is significantly correlated with both depression and anxiety, with an inflection point occurring at a fatigue score of 4 in both models. Once fatigue levels exceed this threshold, the risk of developing these mental health problems increases rapidly, highlighting the compounding effect of moderate to severe fatigue on mental health outcomes. In addition, subgroup analyses indicated that perceived health status and economic status largely moderated this relationship. A recent paper published in The Lancet (36) called for an urgent need to gather information on the mental health impacts of the COVID-19 pandemic on entire populations and the vulnerable groups (e.g., healthcare professionals) in order to address how to reduce the physical and mental health burdens of vulnerable populations under pandemic conditions.

Our study confirmed and extended previous findings that MHPs recovering from COVID-19 infection frequently experienced depression and anxiety (6). Moreover, fatigue as a major symptom of the post-COVID-19 syndrome, is not only common but also leads to the development and exacerbation of psychosocial problems. This is in line with findings that fatigue is a major factor in psychological distress among healthcare workers, especially those working during a pandemic (14, 37).

In contrast to previous research, our study revealed a nonlinear relationship between fatigue and mental health outcomes with an inflection point. Specifically, fatigue levels above 4 led to a significant increase in risk, suggesting that moderate to severe fatigue is a cut-off point beyond which mental health deteriorates more rapidly in this sample. This threshold represents a transition from manageable fatigue that permits normal functioning to more disruptive fatigue that requires compensatory effort and potentially impacts professional performance. The alignment of this inflection point with functional limitations suggests it could serve as an efficient screening threshold for identifying MHPs at elevated risk for developing depression or anxiety. Our finding also aligns with research on chronic fatigue syndrome and long COVID among frontline workers, where excessive fatigue is more likely to trigger negative emotions and contribute to mental health issues such as depression (14, 38).

Several mechanisms may be associated with this nonlinear relationship. Fatigue is strongly linked to cognitive impairments and sleep disturbances, both of which can intensify depression and anxiety (19, 39). As fatigue worsens, these impairments become more pronounced, adding to the psychological burden. Additionally, anxiety-induced physiological arousal can further disrupt sleep, creating a vicious cycle where fatigue and anxiety reinforce each other (40). This may account for the sharp increase in anxiety risk we found at higher fatigue levels. Moreover, fatigue can reduce social engagement and impair work performance, leading to further physical, behavioral and cognitive decline. For MHPs, this can result in unmet professional and personal expectations, contributing to feelings of helplessness and low mood, which exacerbate depression (41).

Our findings also emphasize the moderating role of health and economic status in the relationship between fatigue and depression and anxiety. Participants with poorer health experienced significantly greater increases in depression and anxiety at higher fatigue levels compared to those with better health. This supports existing literature, which shows that individuals with pre-existing health conditions or chronic illnesses are more vulnerable to the adverse mental health effects of fatigue (20). Similarly, participants with lower economic status experienced a significant increase in depression and anxiety as fatigue increased, highlighting the magnifying effect of economic vulnerability on mental health. During the COVID pandemic, healthcare workers were likely to face economic changes (42). Financial insecurity during the pandemic was a significant risk factor for developing depression and anxiety, which may exacerbate the psychological burden of physical fatigue (43). We found that while both depression and anxiety showed similar inflection points, the relationship with perceived economic status appeared stronger for anxiety, suggesting that financial insecurity could particularly exacerbate anxiety when combined with fatigue.

The identification of inflection points in the fatigue-mental health relationship has important implications for clinical practice. Targeted interventions should prioritize MHPs exhibiting moderate to severe fatigue, as they are at a significantly higher risk for depression and anxiety. Early identification of high-risk groups based on fatigue severity allows timely implementation targeted interventions such as cognitive-behavioral therapy (CBT) and fatigue management programs (44). For instance, a study on the mental health of healthcare workers in Wuhan, the first COVID-19 epicenter in China, found that even mild psychological symptoms required mental health support such as remotely delivered psychological therapies and psycho-education, chat lines, digital phenotyping and technologies (45). During the pandemic, emotion and behavioral responses are conceived as part of adaptive responses to extraordinary stress. Thus, psychotherapy treatment based on stress-adaption model may also be helpful in targeting stressors from their roots (46). Additionally, our findings highlight the need for tailored interventions that address the specific needs of MHPs based on their health and economic status. Integrated care models that simultaneously address physical symptoms and mental health issues may be particularly beneficial for those with poor perceived health. MHPs with lower economic status may benefit from additional psychosocial support and financial counseling to alleviate the stressors contributing to their anxiety (42). MHPs who are fatigued may experience burnout, which in turn may compromise the quality of care they are able to provide for their patients (47).

This study has several strengths. By using the RCS model, we gained a more nuanced understanding of the nonlinear relationship between fatigue and depression and anxiety, which allowed us to identify the cut-off point at which fatigue becomes detrimental. Additionally, the large sample size of MHPs recovering from COVID-19 enhances the generalizability of our findings. The inclusion of subgroup analyses based on economic and health status further enriches our understanding of how these factors interact with fatigue to influence mental health. However, some limitations should be noted. First, the cross-sectional study design prevents us from making causal inferences about the relationship between fatigue, depression and anxiety. Second, although fatigue and depression and anxiety were based on validated scales, the use of self-reports may introduce reporting bias. Third, the use of a low screening threshold (PHQ-9 ≥ 5) likely inflated prevalence estimates, although this cutoff value has been widely used in both clinical practice and research. Fourthly, the snowball sampling approach, while necessary due to pandemic restrictions, may introduce potential selection bias. This method may have overrepresented MHPs with more severe symptoms who were motivated to participate. Finally, certain confounding factors related both to fatigue and to depression and anxiety, such as work-related stress or social support, were not recorded in this study.

In conclusion, this study highlights the nonlinear association between physical fatigue and mental health outcomes in MHPs recovering from COVID-19, demonstrating that moderate to severe fatigue is a critical threshold for the disproportionate increase in depression and anxiety. These findings emphasize the importance of early identification and targeted interventions to address fatigue and its mental health consequences, particularly in MHPs with poor health or economic vulnerability. Future longitudinal studies should be conducted to further illustrate the temporal dynamics of the relationship between fatigue, depression and anxiety and develop effective intervention strategies to reduce the psychological burden of fatigue in this vulnerable population.

## Data Availability

The datasets presented in this article are not readily available because the Research Ethics Committee of Beijing Anding Hospital that approved the study prohibits the authors from making publicly available the research dataset of clinical studies.
