# Supplementary material for: The non-linear association of physical fatigue with depression and anxiety among mental health professionals who recovered from COVID-19 infection: a national survey in China
Source: Front Psychiatry. 2025 Aug 22;16:1610910. doi: 10.3389/fpsyt.2025.1610910 (PMC12411462; doi:10.3389/fpsyt.2025.1610910)
Supplement: Supplementary file 1 [file Supplementaryfile1.docx]

**Supplemental materials**

**Supplementary Table 1.** Variance–Inflation Factors (VIF) for Covariates in Multivariate Logistic-Regression Models

**Figure S1.** Nonlinear association between depression (a), anxiety (b) and physical fatigue, stratified by gender

**Figure S2.** Nonlinear association between depression (a), anxiety (b) and physical fatigue, stratified by age group

**Figure S3.** Nonlinear association between depression (a), anxiety (b) and physical fatigue, stratified by marital status

**Figure S4.** Nonlinear association between depression (a), anxiety (b) and physical fatigue, stratified by COVID-19 quarantine experience

**Figure S5.** Nonlinear association between depression (a), anxiety (b) and physical fatigue, stratified by smoking status

**Figure S6.** Nonlinear association between depression (a), anxiety (b) and physical fatigue, stratified by drinking status

**Supplementary Table 1.** Variance–Inflation Factors (VIF) for Covariates in Multivariate Logistic-Regression Models

| **Variables** | **Depression Model VIF** | **Anxiety Model VIF** |
| --- | --- | --- |
| Physical Fatigue | 1.22 | 1.22 |
| Age | 7.43 | 7.44 |
| Work years | 7.43 | 7.47 |
| QOL | 1.31 | 1.31 |
| Male | - | 1.56 |
| Perceived economic status | 1.07 | 1.07 |
| Perceived health status | 1.18 | 1.18 |
| Being quarantined during the COVID-19 pandemic | 1.01 | 1.01 |
| Smoking | 1.14 | 1.52 |
| Drinking | 1.14 | 1.19 |


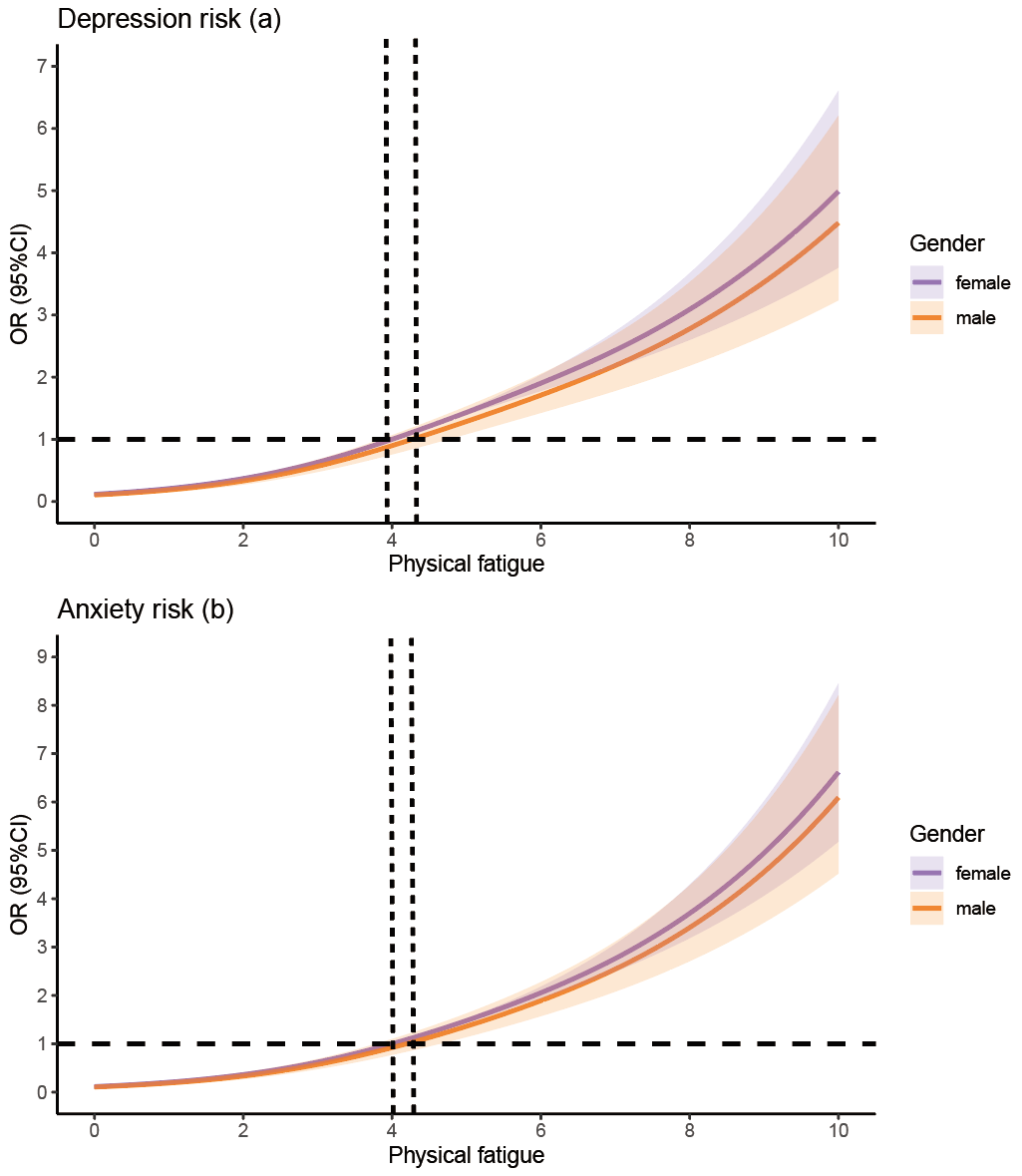


**Figure S1.** Nonlinear association between depression (a), anxiety (b) and physical fatigue, stratified by gender. Vertical lines indicate inflection points.


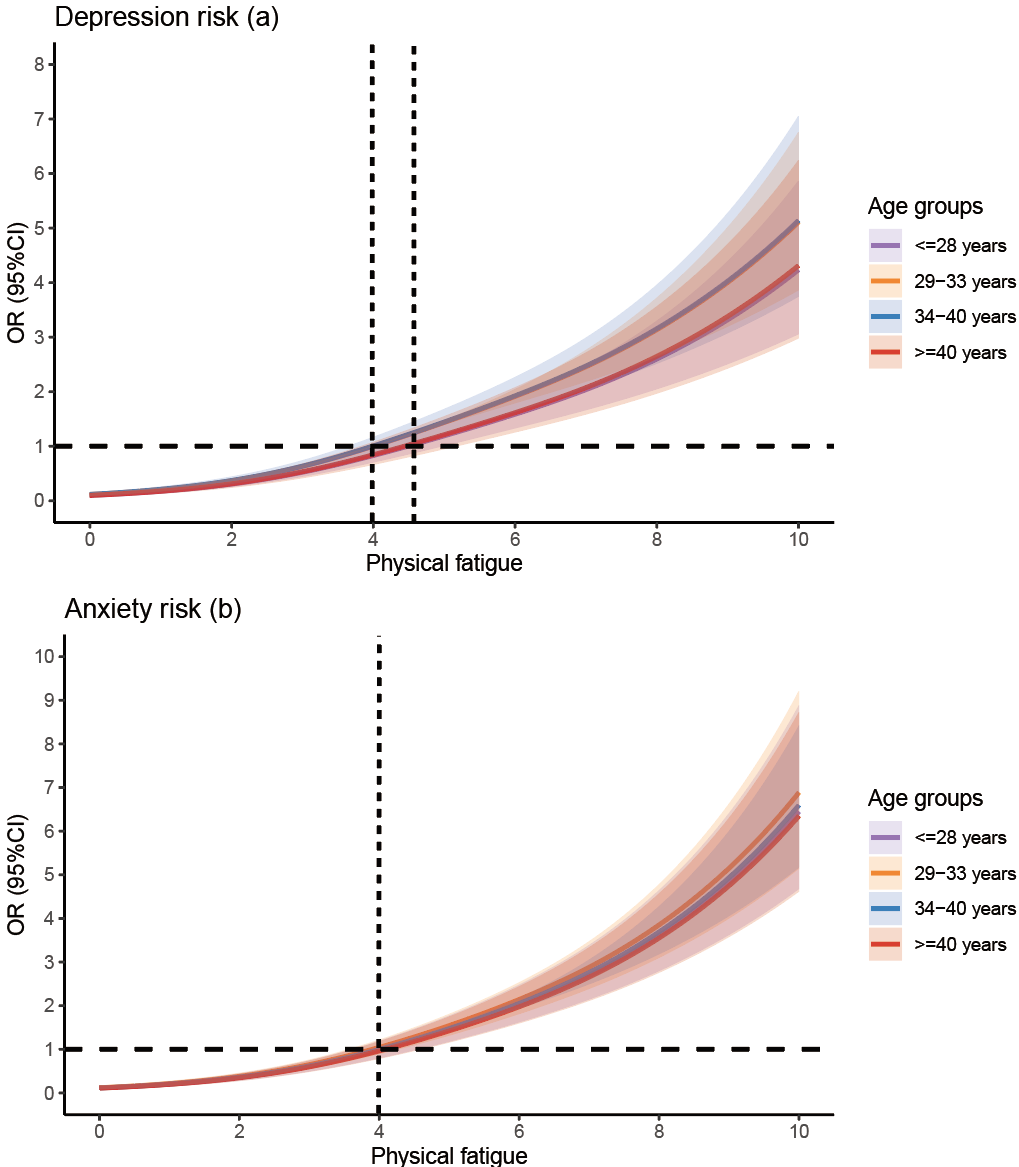


**Figure S2.** Nonlinear association between depression (a), anxiety (b) and physical fatigue, stratified by age group. Vertical lines indicate inflection points.


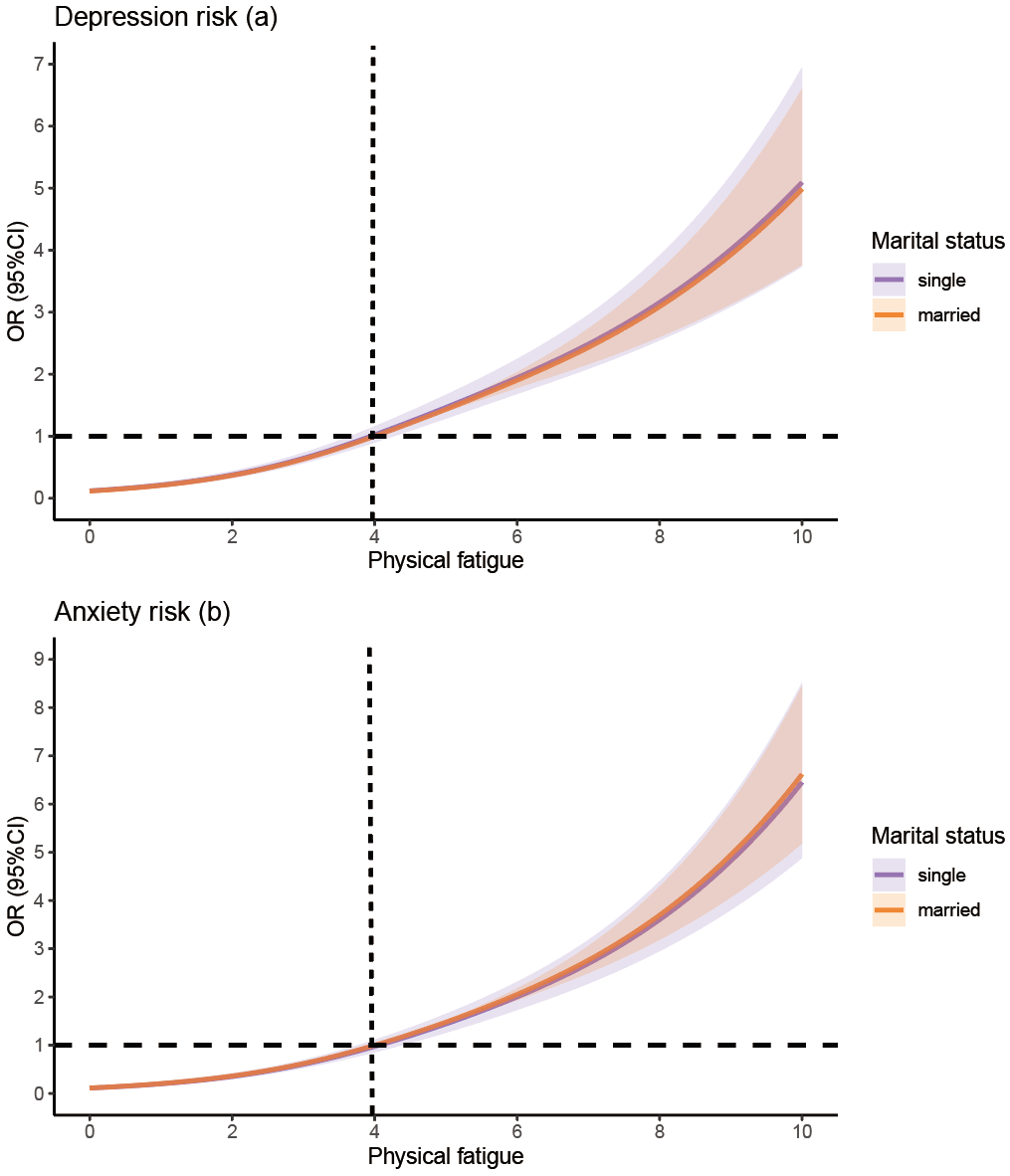


**Figure S3.** Nonlinear association between depression (a), anxiety (b) and physical fatigue, stratified by marital status. Vertical lines indicate inflection points.


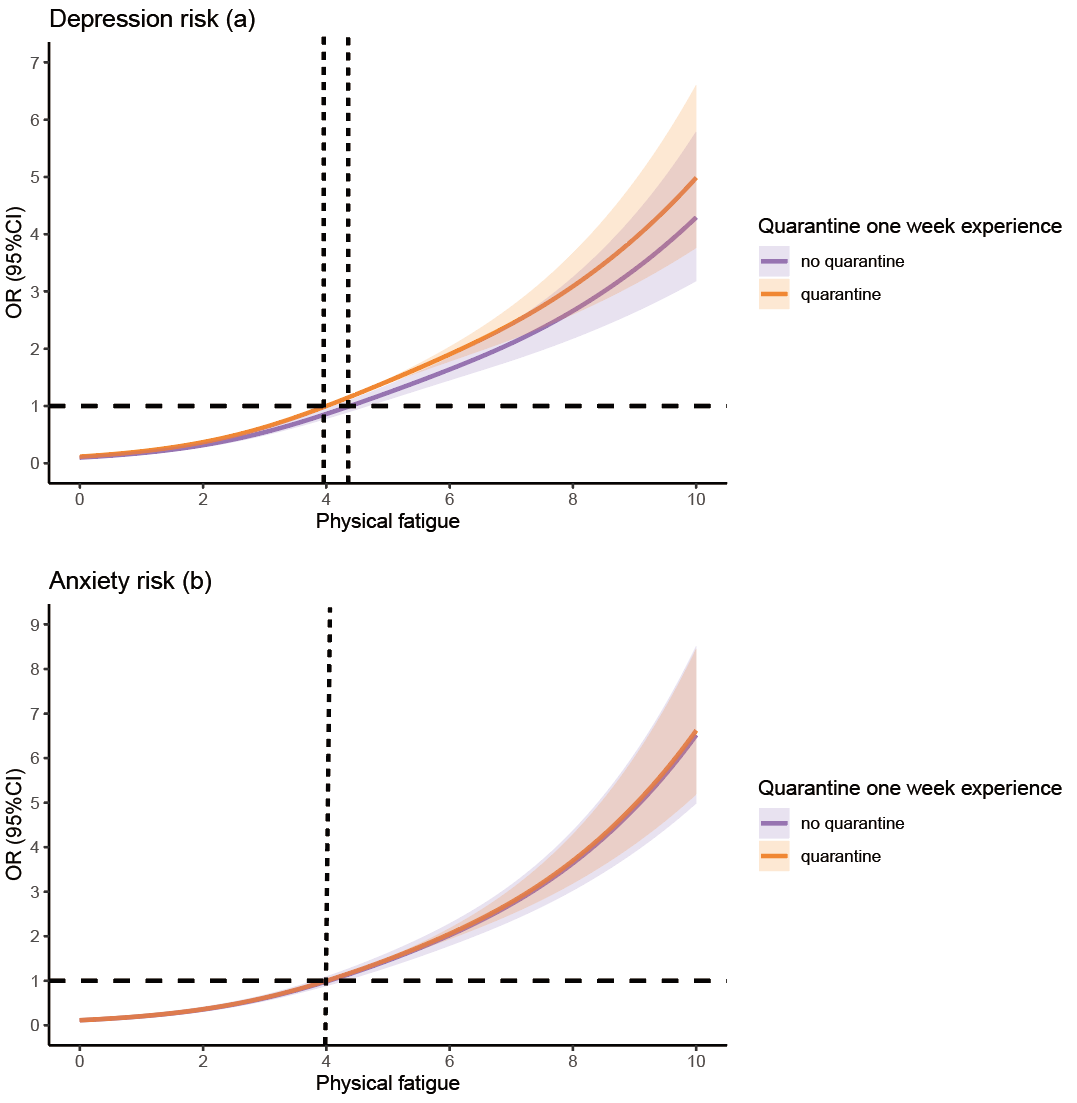


**Figure S4.** Nonlinear association between depression (a), anxiety (b) and physical fatigue, stratified by COVID-19 quarantine experience. Vertical lines indicate inflection points.


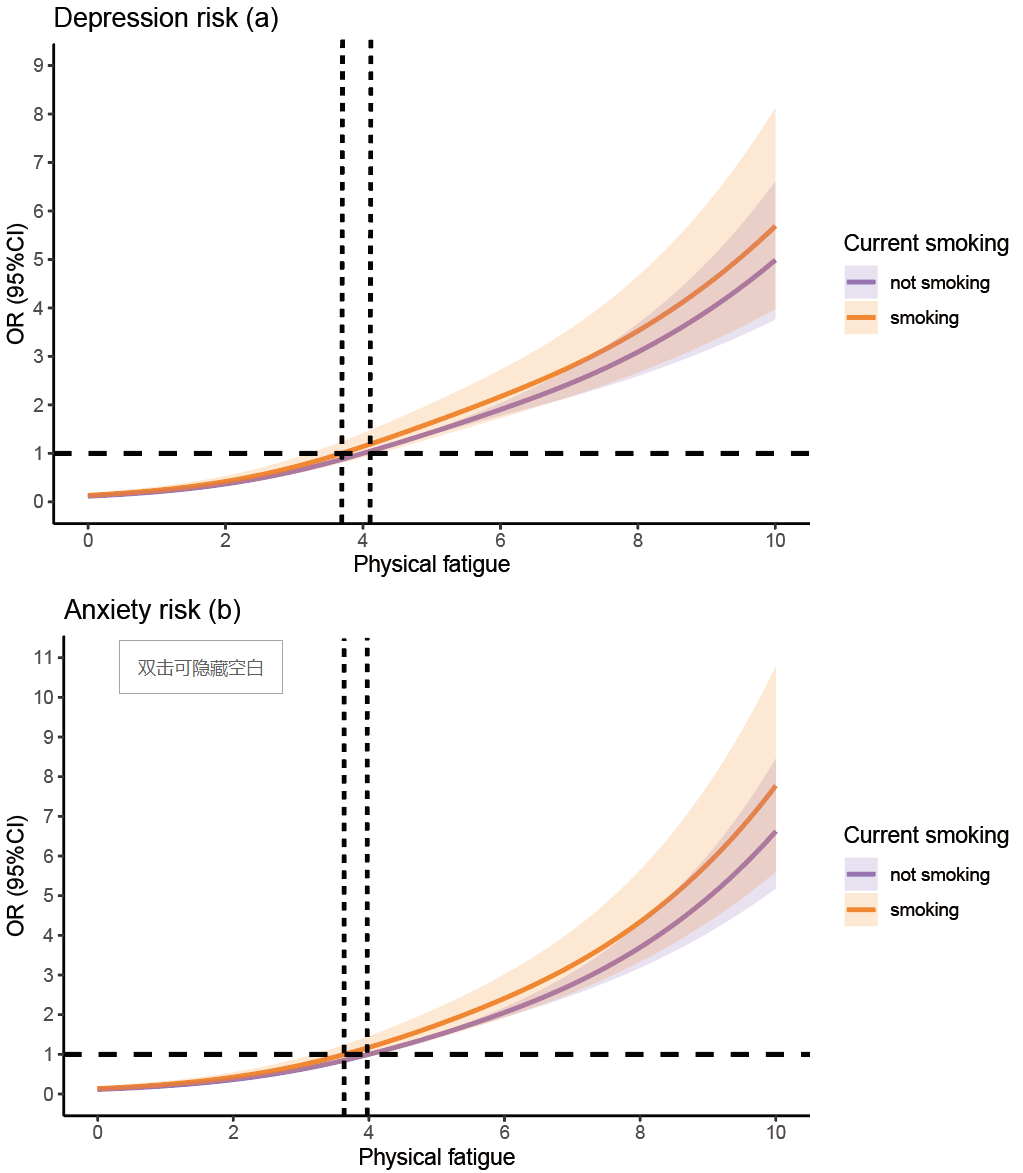


**Figure S5.** Nonlinear association between depression (a), anxiety (b) and physical fatigue, stratified by smoking status. Vertical lines indicate inflection points.


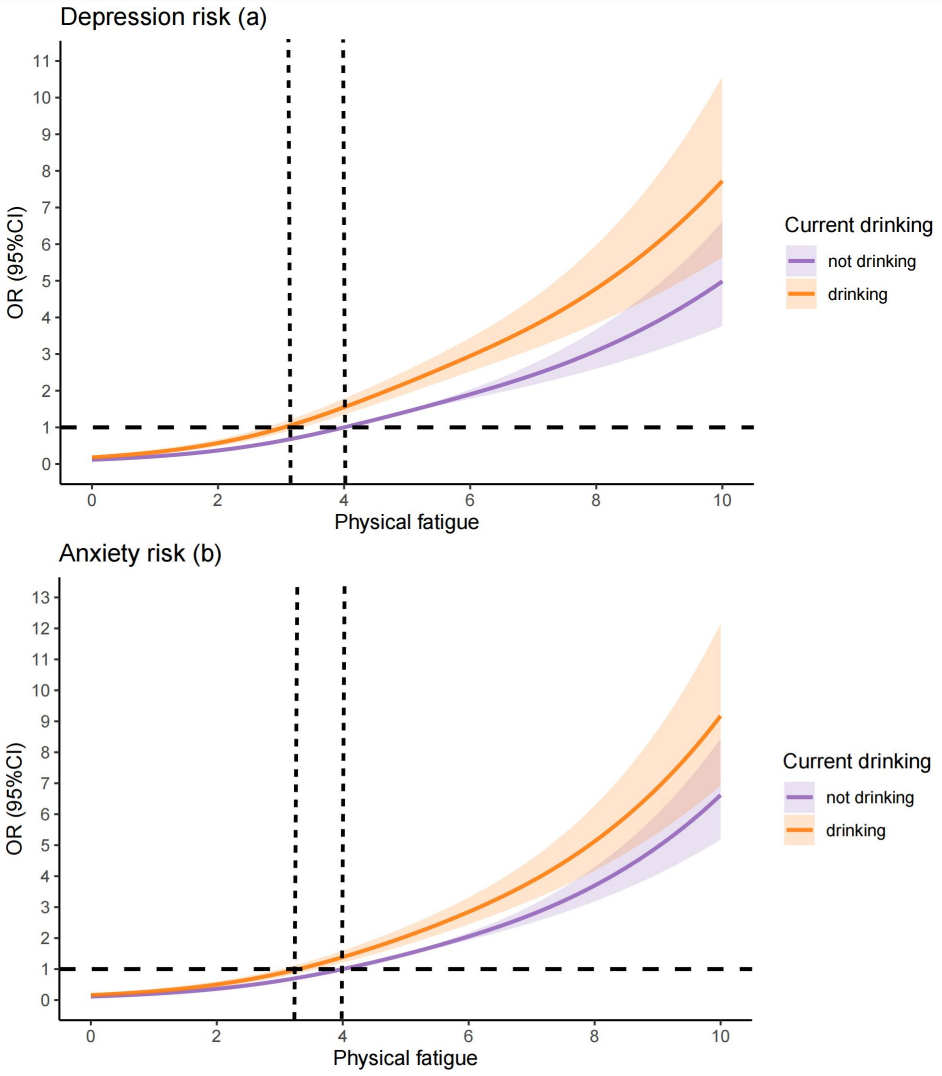


**Figure S6.** Nonlinear association between depression (a), anxiety (b) and physical fatigue, stratified by drinking status. Vertical lines indicate inflection points.
